# Absence of a specific radiation signature in post-Chernobyl thyroid cancers

**DOI:** 10.1038/sj.bjc.6602521

**Published:** 2005-04-05

**Authors:** V Detours, S Wattel, D Venet, N Hutsebaut, T Bogdanova, M D Tronko, J E Dumont, B Franc, G Thomas, C Maenhaut

**Affiliations:** 1 Institute of Interdisciplinary Research, School of Medicine, Free University of Brussels, Campus Erasme, route de Lennik 808, B-1070 Brussels, Belgium; 2 Institute of Endocrinology and Metabolism, 04114 Kiev, Ukraine; 3 Service d'Anatomie et de Cytologie Pathologiques, Hôpital A Paré (AP-HP), Université de Versailles, St Quentin en Yvelines, France; 4 South West Wales Cancer Institute/Swansea Clinical School, Singleton Hospital, Sketty Lane, Swansea SA2 8QA, UK

**Keywords:** thyroid, papillary, post-Chernobyl, microarray

## Abstract

Thyroid cancers have been the main medical consequence of the Chernobyl accident. On the basis of their pathological features and of the fact that a large proportion of them demonstrate RET-PTC translocations, these cancers are considered as similar to classical sporadic papillary carcinomas, although molecular alterations differ between both tumours. We analysed gene expression in post-Chernobyl cancers, sporadic papillary carcinomas and compared to autonomous adenomas used as controls. Unsupervised clustering of these data did not distinguish between the cancers, but separates both cancers from adenomas. No gene signature separating sporadic from post-Chernobyl PTC (chPTC) could be found using supervised and unsupervised classification methods although such a signature is demonstrated for cancers and adenomas. Furthermore, we demonstrate that pooled RNA from sporadic and chPTC are as strongly correlated as two independent sporadic PTC pools, one from Europe, one from the US involving patients not exposed to Chernobyl radiations. This result relies on cDNA and Affymetrix microarrays. Thus, platform-specific artifacts are controlled for. Our findings suggest the absence of a radiation fingerprint in the chPTC and support the concept that post-Chernobyl cancer data, for which the cancer-causing event and its date are known, are a unique source of information to study naturally occurring papillary carcinomas.

Thyroid cancer is the most common form of solid neoplasm associated with radiation exposure. There has been a considerable increase in thyroid cancer after the Chernobyl nuclear power plant accident, particularly in subjects who were in childhood or adolescence at the time of exposure. This is due to the exposure of the population to I^131^ and shorter lived radioisotopes of iodine. These thyroid cancers have been identified as papillary carcinomas on the basis of their pathology (Robbins and Schneider, 1998; Shibata *et al*, 2001). Thyroid cancers related to external radiation in the US have been diagnosed as papillary carcinomas on the same basis (Roudebush *et al*, 1978). As the incidence of thyroid cancer in young children and adolescents is very low in unexposed populations, the majority of thyroid cancers occurring in this population can be ascribed to a direct result of exposure to radiation (Malone *et al*, 1991). The Chernobyl accident therefore provides a unique opportunity to characterize radiation-induced thyroid cancer.

RET proto-oncogene rearrangements (frequency: ∼30% in adult PTC) and BRAF somatic mutations (frequency: 36–69% in adult PTC) (Cohen *et al*, 2003; Kimura *et al*, 2003; Soares *et al*, 2003) represent the most common genetic alterations found in sporadic, naturally occurring, papillary thyroid carcinomas (PTC). RET rearrangements result from the fusion of the RET tyrosine kinase domain with the N-terminus part of different proteins, creating chimeric oncogenes with constitutive activity, named RET/PTC (Jhiang, 2000). At least 15 different RET/PTC variants have been described so far involving rearrangement with 10 different genes (Tallini and Asa, 2001).

Post-Chernobyl PTC (chPTC) have shown so far a higher frequency of rearrangement of the RET/PTC oncogene (Nikiforov *et al*, 1997) and lower frequency of BRAF mutation (Nikiforova *et al*, 2004) than is observed in adult PTC. This may be linked to the particular effectiveness of radiation in causing double-strand breaks and gene rearrangements, rather than point mutations. RET rearrangements, which would be the direct results of double-strand breaks repairs and which are thus presumed to be early events in thyroid tumorigenesis, are found in about 60% of these tumours (Fugazzola *et al*, 1995; Klugbauer *et al*, 1995; Bounacer *et al*, 1997). The majority of these are RET/PTC3 rearrangements and are associated with a solid/follicular subtype of PTC, while the less frequent RET/PTC1 rearrangement is associated with the classic and diffuse sclerosing variants (Thomas *et al*, 1999; Santoro *et al*, 2000). While the presence of RET/PTC translocations in a sizeable proportion of post-Chernobyl thyroid cancers and sporadic papillary carcinomas is an argument in favour of the identity of both types of tumours, the different proportions of the types of translocation could suggest a different molecular phenotype.

Gene expression profiling of clinical samples by microarray offers unprecedented opportunities to define molecular signatures of the pathology of tissue samples. In this work, we show that, while hyperfunctioning thyroid adenomas and papillary carcinomas are easily distinguishable on the basis of their gene expression patterns, post-Chernobyl, radiation-induced PTC have the same molecular phenotype as sporadic PTC (sPTC) from Belgium, France and the US. This suggests that a molecular signature for radiation-induced thyroid cancer is very unprobable.

## MATERIALS AND METHODS

### Tissue samples

Human thyroid samples were obtained after surgical resection for thyroid tumours: 8 sPTC, 12 chPTC, and 13 autonomously functioning adenomas. The tissues were immediately kept at 4°C, dissected, snap-frozen in liquid nitrogen and then stored at −80°C till processing. The protocol received approval from the ethics committees of the institutions.

### RNA purification

The frozen tissues were reduced to powder in liquid nitrogen, and total RNA was extracted using a Trizol reagent kit (Life Technologies Inc., CA, USA) followed by purification on Qiagen RNeasy columns. The concentration of the RNA was assessed spectrophotometrically and its integrity was verified by visualisation of intact 18S and 28S ribosomal RNA bands after gel migration. Samples from post-Chernobyl thyroid cancers were obtained via the Chernobyl Tissue Bank (http://www.chernobyltissuebank .com).

The presence of RET/PTC gene rearrangement was analysed by RT–PCR as described by Santoro *et al* (2000), and the presence of mutated BRAF^T1796A^ was detected as described by Nikiforova *et al* (2003).

### Micromax cDNA microarray hybridisation

Tumour RNA extracts were hybridised together with patient-matched nontumoral thyroid tissues on *Micromax Human cDNA microarray system* (Perkin-Elmer, Wellesley, MA, USA), containing 2400 known human cDNA, mapping to ∼2100 UniGene (build 160) clusters. All hybridisations were replicated with fluorescent dyes swapped between tumour/nontumoral pairs. Total RNA (1–1.5 *μ*g/slide) was labelled with fluorescein or biotin according to the manufacturer's instructions (Micromax labelling kit, Perkin-Elmer Life Science). After resin column purification (Microcon YM-100, Millipore, Billerica, MA, USA) and ethanol precipitation, each labelled target was resuspended in 30 *μ*l hybridisation buffer containing 50% formamide, 7 × SSPE (20 × SSPE=3.6 M NaCl, 0.2 M sodium phosphate, pH 8.3, 20 mM EDTA), 5 × Denhardt's (0.1% Ficoll, 0.1% polyvinylpyrrolidone), 0.5% SDS, along with 10 *μ*g of polydA (Research Genetics, Groningen, The Netherlands) and 10 *μ*g of Cot-1 DNA (Gibco Life Sciences, Paisley, UK), which were added in order to reduce cross-hybridisation and nonspecific hybridisation. The arrays were prehybridised at 42° for 30 min in 5 × Denhardt, 7 × SSPE, 0.5% SDS and 1% bovine serum albumin. Prior to hybridisation, carried out overnight at 42°C in a Corning hybridisation chamber, the labelled cDNA was denatured for 3 min at 95°C and then incubated at 42°C for 20 min. Following the hybridisation, the arrays were washed twice with 0.1 × SSC (1 × SSC=0.15 M NaCl, 0.015 M sodium citrate), 0.1% SDS for 10 min and three times with 0.1 × SSC for 10 min. Fluorescein and biotin labelled cDNAs were sequentially detected according to the tyramide signal amplification (TSA) process, following the manufacturer's instructions: hybridisation signal from fluorescein labelled cDNA was amplified with antifluorescein antibody coupled to horseradish peroxidase (HRP) and cyanine 3 tyramide, while hybridisation signal from biotin labelled cDNA was amplified with streptavidin, HRP-coupled, and cyanine 5 tyramide.

### Preprocessing of microarray data

Micromax arrays were scanned on a GMS 418 array scanner (Affymetrix, Santa Clara, CA, USA). Scans were quantified at multiple gains with ImaGene 4.0 (Biodiscovery, Marina del Rey, CA, USA). Each experiment was performed at least twice with dye swap. Quantification performed at different gains were merged in order to better capture the full dynamic range of fluorescence intensities. The scan merging algorithm is implemented in the MatArray toolbox (Venet, 2003). Following background substraction, the resulting expression measurements were corrected for spatial and intensity biases using algorithms from the MatArray toolbox (Venet, 2003). Corrected log-ratios of on-slide and dye-swap replicates were averaged. cDNA probes mapping to the same LocusLink ID were also averaged.

### Unsupervised classification

Hierarchical clustering was performed on the basis of the 50% genes with the highest median intensity, using the Pearson correlation distance and Ward linkage as implemented in the mva library of the R 1.9.0 statistical package (R development Core Team, 2004). Multidimensional scaling was performed on all genes. We used the Pearson correlation distance, and the isoMDS function from the MASS library (Venables and Ripley, 2002) for R. The R 1.9.0 implementation of *k*-mean was used. The algorithm was run 100 times with random initial conditions, and with two centres, on the full data set. Classification results were then derived by majority voting over the 100 runs. The same procedure was repeated on PTC alone to search for a sporadic/post-Chernobyl separation.

### Detection of differentially expressed genes

Differentially expressed genes were searched for with the significance analysis of microarray (SAM) procedure (Tusher *et al*, 2001) as implemented in the siggenes library (Schwender, 2003) for R. This permutation-based method avoids parametric hypotheses and handles multiple testing issues inherent to microarray data. A total of 1000 random permutations or the class labels were examined in each run of SAM.

### Supervised classification

Expression signatures separating tumour types were found with an algorithm akin to diagonal linear discriminants introduced by Golub *et al* (1999), and discussed by Dudoit *et al* (2002). Discriminating signatures were assessed by cross-validation. Two-third of the samples were selected randomly and used to build the signature. The remaining 1/3 samples were used to test its classification performances. Classification errors were averaged over 100 runs of this protocol. In order to compute an upper bound for the *P*-values of the classification error, the entire cross-validation procedure was repeated 100 times on the data with randomly permuted class labels. In order to check that the three-fold cross-validation scheme was not too stringent to obtain result for the sporadic/post-Chernobyl cancer classification, we also run leave-one-out cross-validation for this task. Again, sporadic and post-Chernobyl cancers could not be separated. Supervised classification was also performed using the Pelora algorithm (Dettling and Bühlmann, 2004) as implemented in the supclust library for R, with parameters *λ*=1/16 and *n*=10 groups, and the cross-validation protocol with 2/3–1/3 training-testing split described above.

### Correlation analysis

Tumour RNA extracts from 14 sPTC were pooled and hybridised with their respective control pools on dual channel Human 5k microarrays (Flanders Interuniversity Institute for Biotechnology, Leuven, Belgium, http://www.microarrays.be). The Human 5k slide set includes four slides onto which 18 442 ESTs representing 13 000 human genes are spotted in duplicate. A dye-swap hybridisation was performed. A similar protocol was applied to RNA extracts from 13 chPTC and from five autonomously functioning adenoma. The chPTC pool, however, was hybridised on Human 21k microarrays (Flanders Interuniversity Institute for Biotechnology, Leuven, Belgium), which include 21 000 ESTs representing 16 000 genes. All microarrays were processed according to Flanders Interuniversity Institute for Biotechnology's standard protocol (http://www.microarray.be/servi ce.htm). Background-corrected, intensity-normalised, data were used in subsequent analysis.

We downloaded the raw Affymetrix HGU95 data (CEL files, http://thinker.med.ohio-state. edu/ptc/) of Huang *et al* (2001) and normalised them with the RMA method (Irizarry *et al*, 2003) as implemented in the ‘rma’ function of the Bioconductor package (version 1.4, http://www.bioconductor.org/) for the R statistical language. Log-ratios were computed and averaged over all eight patients of Huang's study. For each data set, further averaging was performed over probe sets mapping to a same UniGene cluster. Pearson correlations between averaged log-ratios were then computed over the 6425 UniGene clusters represented on the HGU95, Human 5k, and Human 21k platforms. Pearson correlations were then computed on the resulting log-ratios.

### Real-time RT–PCR

5 *μ*g of total RNA was treated with 5 U DNAseI (Life Technologies) during 15 min at room temperature in a total volume of 50 *μ*l in order to remove possible genomic DNA contamination. The whole reaction volume was then subjected to reverse transcription using Superscript II (Life Technologies) as described by the manufacturer, in a final volume of 100 *μ*l (0.05 *μ*g cDNA/100 *μ*l), and the cDNA obtained was diluted five times. Oligonucleotide sequences (primers and probes) corresponding to the selected transcripts were designed using Primer Express software (Applied Biosystems, CA, USA). The nucleotide sequence of the primers and the probes are available upon request. The Taqman probes carry a 5′ reporter label (FAM) and a 3′ dark quencher (Dabcyl). Relative quantifications of gene expression were performed with the ABI Prism 7700 Sequence Detection System (AB/Perkin-Elmer) using 2 *μ*l of the diluted cDNA product as template for each reaction, 300 nM of a forward and a reverse primer, 100 nM probe and the quantitative PCR mastermix with ROX as the passive reference (Eurogentec, Liège, Belgium) in a total volume of 30 *μ*l. The mastermix contains a hotstart enzyme, which is activated by heating for 10 min at 95°C. Amplification was carried out using 40 PCR cycles consisting of a two-step procedure, 60 s at 60°C and 60 s at 95°C. The threshold cycle (Ct) was determined as the fractional cycle number at which the amount of amplified target reached a threshold, which was fixed at 0.06, where all the amplification curves were above background fluorescence and still in exponential phase. For calculating the expression level, we used a mathematical model developed by Pfaffl (2001), which determines the relative quantification of a target gene in comparison to a reference gene (PBGD) based on the PCR efficiencies of each gene and the Ct deviation of an unknown sample *vs* a control (in our case tumour *vs* nontumoral sample): 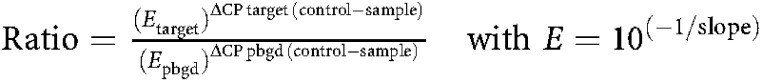


All the real-time RT–PCR experiments were performed in duplicate or triplicate.

## RESULTS

### Different clustering algorithms do not separate sporadic and chPTC, but do separate autonomous adenomas (AAs) from PTC

**Figure 1:**
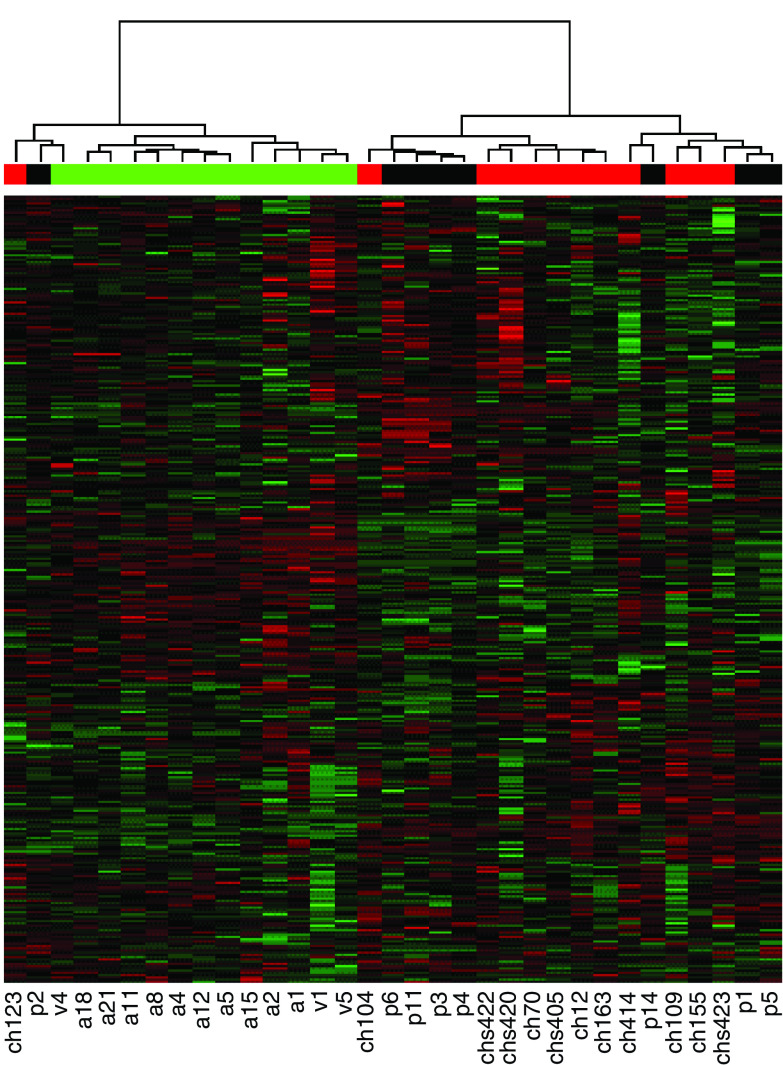
Hierarchical clustering of the microarray data from 13 autonomous adenomas (noted a or v) and 20 PTC (p: sporadic, ch: post-Chernobyl PTC). The colour bar below the dendogram depicts tumour types: green, autonomous adenomas; black, sporadic PTC; red, post-Chernobyl PTC. Each row represents a cDNA clone and each column a tumour RNA sample. Red indicates upregulation, green downregulation, and black no change. The normalisation and clustering procedures are described in the Materials and Methods section.

**Figure 2:**
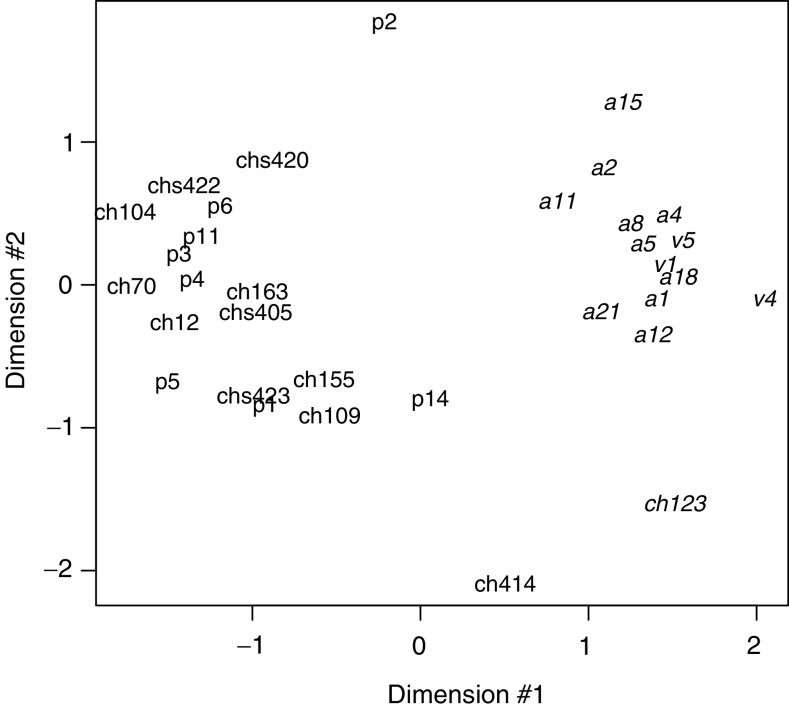
Samples were collapsed from the 2400 dimensions gene expression space into two dimensions using multidimensional scaling, a mathematical transformation that preserves intersample distance relationship. The distance metrics used here is Pearson correlation. Autonomous adenomas are in italic, sporadic PTC in bold and post-Chernobyl PTC in standard characters. The intersample distances in the two-dimensional embedding are expressed in arbitrary units, but are proportional on average to the gene space correlation distances (not shown).

Microarray experiments were performed using the Micromax cDNA system from Perkin-Elmer, containing 2400 known human cDNA spotted in duplicate. For each sample, experiments were duplicated with dye swap, and the averages of the intensity ratios (tumour/control) resulting from each dye combination were calculated (see Materials and Methods). In total, 20 cases of PTC were studied: among the latter, eight were cases with no history of radiation exposure, treated surgically at the Erasmus Hospital (Free University of Brussels, Belgium), or at the A Paré Hospital (AP-HP, Boulogne, France), and 12 samples were chPTC obtained from the Chernobyl Tissue Bank. The clinical parameters and gene alterations, including BRAF^T1796A^ mutation and RET/PTC gene rearrangements, for each of the cases examined in this study are summarised in Table 1. In addition, 13 autonomous thyroid adenomas (AA) were included as controls, that is, to demonstrate that the methods applied in the paper do detect differences between differing tumour types. Hierarchical clustering of the samples, performed using the 50% most expressed genes, separated papillary carcinomas from AAs except for two samples, p2 and ch123 (Figure 1). However, no such separation was observed between the PTC of different aetiology: there was no clustering of the sPTC separately from chPTC. In addition, chPTC could not be segregated into those which harboured a RET/PTC rearrangement and those that did not. Similar results were obtained using *k*-mean classification (not shown, see Materials and Methods), and were further confirmed with multidimensional scaling on the basis of all 2400 genes, as shown in Figure 2. Multidimensional scaling (Venables and Ripley, 2002) collapses the 2400 dimensions gene expression data into two dimensions while preserving the distance relationship between samples. Its strength over hierarchical clustering is that it is a continuous representation of the data faithful to the continuous nature of the gene expression space. Adenomas are tightly grouped in the two-dimensional embedding, and well separated from the PTC. Sporadic and chPTC are mixed up. Samples ch123 and p2, which were misclassified by hierarchical clustering and by *k*-mean are apart from the PTC group, and from the adenomas group. The strikingly different patterns of gene expression in AAs and papillary carcinomas are entirely consistent with the known mutually exclusive genotypes, which often cause these lesions: constitutive activation of the thyrotropin (TSH)/cAMP and of the RET/PTC–RAS–RAF–MAP kinase pathways respectively.

**Figure 3:**
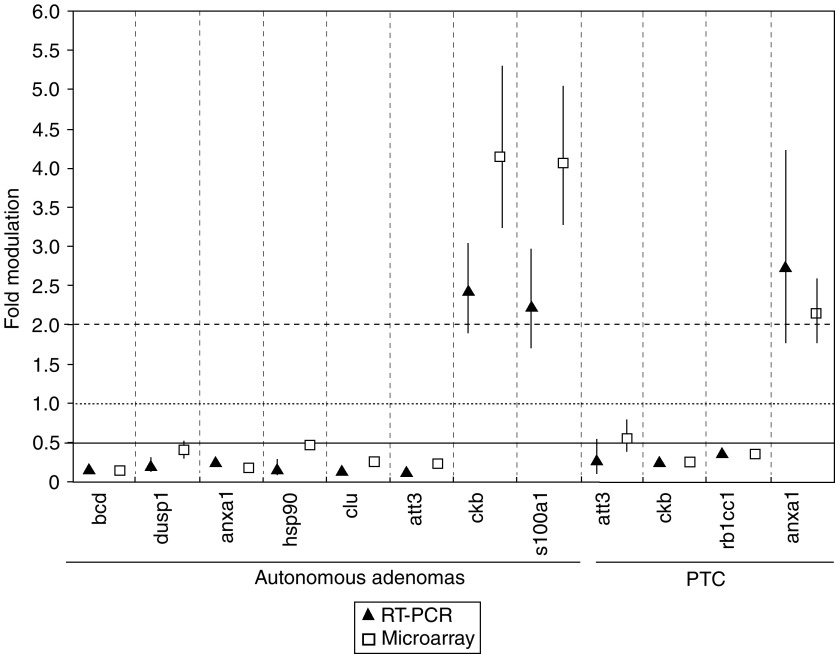
Validation of microarray data by real-time RT–PCR analysis of selected genes, in PTC and hyperfunctioning autonomous adenomas samples (±s.e.m.).

Several of our microarray results are in accordance with previous data obtained in our group by Northern blot analysis (Deleu *et al*, 2000), or with data from the litterature (Huang *et al*, 2001; Wasenius *et al*, 2003; Eszlinger *et al*, 2004; Finley *et al*, 2004a, 2004b). In addition, we validated our microarray data by evaluating the expression of selected transcripts using real-time RT–PCR (Figure 3).

### SAM analysis cannot differentiate sporadic from chPTC, but finds genes differing between adenomas and PTC

Significance analysis of microarray (SAM) (Tusher *et al*, 2001) is a procedure that detects differentially expressed genes without making hypotheses on the distribution of expression levels, and with a rigorous handling of the fact that thousands of hypotheses are being tested at once in a microarray analysis. As a statistically sound alternative to the *ad hoc* two-fold change threshold used in many publications to declare a gene differentially expressed, SAM uses the false discovery rate (FDR), that is, an estimate of the probability that the genes found to be differentially expressed are false positives. When comparing adenoma and PTC, SAM detected 168 differentially expressed genes in 2400 at an FDR of 1%. When comparing sporadic and chPTC, no differentially expressed genes could be found even using a less stringent FDR of 5%.

### Supervised classification fails to find a signature separating sporadic and chPTC

None of the genes detected by SAM could perfectly separate adenomas from PTC when considered alone. Hence, we searched a set of genes leading to a more accurate adenoma/PTC separation when considered collectively. The classification method proposed by Golub *et al* (1999) finds genes that show large and consistent expression differences between two groups of samples (see Materials and Methods). A set of such genes is called a signature. We searched for signatures ranging in size from 1 to 1000 genes. The predictive value of the signatures for classification was measured for each size through cross-validation (see Materials and Methods). The best signatures were composed of six genes, and resulted in a misclassification rate of 7% (*P*<0.01). These six genes were also identified by SAM with an FDR <0.001. Smaller signatures lead to higher error rates. Only the average behaviour of the genes composing the signature indicates a clear adenoma/PTC distinction. Neither the procedure of Golub *et al* (1999), nor Pelora (Dettling and Bühlmann, 2004) could find a signature classifying sporadic and chPTC better than a random class assignation. To check that we found of a signature separating adenomas from PTC, but no signature separating sporadic from chPTC, because fewer samples were used in this latter classification task – adenomas are excluded – we ran the adenoma/PTC classification using sPTC only. Error rates as low as 3% (*P*<0.01) were obtained with a 36 genes signature.

### Cross-platform control on the basis of 6425 genes shows that sporadic and chPTC expression data sets are as highly correlated as two independent sPTC data sets

The 2400 randomly selected cDNAs present on the Micromax slides cover about 8% of the potential human transcriptome. We therefore wondered whether the similarity between sporadic and chPTC would hold for a larger number of genes. In addition, published comparison of different microarray platforms suggests strong platform-specific effects (Detours *et al*, 2003), raising the possibility that our results relying on Micromax arrays may not be reproducible on other platforms. We addressed these issues by comparing PTC data generated on different platforms in our laboratory and in another laboratory, which uses completely independent protocols to select tumours and to process samples. The first additional data set was produced in-house as follow: 14 sPTC RNA samples were pooled and hybridised together with a patient-matched nontumour thyroid tissue pool on Human 5k dual channels microarrays from the Flanders Interuniversity Institute for Biotechnology (VIB, Leuven) covering about 16 000 genes. A similar protocol was applied to 13 chPTC and to five AA samples using VIB's Human 21k and Human 5k arrays, respectively. The second data set relies on completely independent technologies and biological material. Huang *et al* (2001) hybridised eight PTC and eight patients-matched nontumoral tissues on Affymetrix (Affx) HGU95 oligonucleotide microarrays. The Affymetrix probe design is unlikely to share the same crossreactivity patterns as those of the cDNA platforms we used. In addition, Huang's samples were selected and processed in the United States, independently from ours. After normalisation, intensity log-ratios were averaged over the eight patients in order to simulate a pool comparable to our in-house, VIB, pool data.

Correlations between averaged log-ratios were then computed over the 6425 genes represented on the HGU95, VIB 5k and VIB 21k platforms. The results are shown in Table 2. Correlations between patient-averaged Affx PTC data and our three RNA pools data sets were 0.49 for Affx PTC *vs* our sPTC, 0.64 for Affx PTC *vs* chPTC. Thus, the Affx PTC and chPTC data sets are at least as correlated as the two independent sPTC data sets. By contrast, correlation was only 0.03 between the Affx PTC and the adenoma data sets. This control demonstrates that the high correlation between the PTC data sets is not a statistical or a technology-related artifact. The higher correlation with chPTC may be due to the fact that this pool was hybridised on Human 21k slides, which are based on a more recent and more accurate technology than that of the Human 5k slides used for sPTC. Considering only the 50% most expressed genes in the calculation increased the correlation between the two sPTC data sets and the correlation between Huang *et al* data and our chPTC data, but it did not increase the correlation between the Huang *et al* data set and our adenoma data.

## DISCUSSION

Our results show that (1) RNA profiles from sporadic and chPTC do not permit identification of a molecular signature specific for radiation-induced or sPTC, on the basis of the expression of 2400 randomly selected genes, while AAs and PTC show distinct gene expression signatures and (2) sporadic and chPTC pools are as strongly correlated as two independently derived PTC pools on the basis of 6425 genes. Huang's (Huang *et al*, 2001) data rely on independent tissue samples selected by different pathologists, processed in an independent laboratory, and hybridised with a different microarray technology. Thus, artefacts pertaining to these matters are controlled for.

Cross-platform integration is necessary if the full potential of high-throughput expression technologies is to be realised (Detours *et al*, 2003; Moreau *et al*, 2003). The similarities between our dual channel cDNA data and Huang's single channel oligonucleotide data contrast with a widely cited paper reporting that these technologies may not be comparable (Kuo *et al*, 2002). According to this report, Affymetrix and cDNA microarrays measurements of gene expression in NCI 60 cancer cell lines are poorly correlated. This discrepancy could be due to the fact that we compare log-ratios with log-ratios while Kuo *et al* (2002) compares MAS 4.0 average difference (i.e. expression level computed by the Affymetrix microarray analysis software) with either data from a single cDNA channel, or with log-ratios of the two cDNA channels. In addition, we found that oligonucleotide and cDNA data were comparable only after appropriate data normalisation. Using the original normalisation of Huang, based on the method of Li and Wong (2001) led to weak correlations with cDNA data (not shown). By contrast, investigation is limited to MAS 4.0 expression measures in Kuo *et al* paper and cDNA data were not normalised.

One caveat of this work is the limited number of genes investigated in the multiple samples analysis, and the fact that our second study, extended on more genes, has been performed with pooled samples. However, we may consider that, when these data are taken together, the results of these studies are conclusive. In addition, preliminary results using 16 000 genes arrays are in accordance with the data presented here.

The clinical features of French or Italian sporadic papillary carcinomas in children were similar to those of children's post-Chernobyl carcinomas (Leenhardt and Aurengo, 2000). Similarly, thyroid cancers in comparably young children in the US who had been exposed to external head and neck irradiation behaved clinically as those of nonexposed children (Becker *et al*, 1996). This led to the suggestion that radiation-induced thyroid tumours should be treated in the same way as nonradiation-induced tumours (Schneider and Fogelfeld, 1997). This similarity between sporadic and radiation-induced PTC might be explained by the hypothesis that part or most of the sporadic papillary cancers encountered in the normal population might be due to natural background irradiation (DeGroot, 1989). However, the proportion of such cancers due to the Chernobyl accident in Western countries is certainly negligible (Malone *et al*, 1991), and the possibility of Chernobyl radiation is ruled out for the sPTC from the US patients considered in this study. The similarity of gene expression profiles of post-Chernobyl, Western European, and US PTC validates quantitatively the identification of these tumours by pathologists on the basis of their qualitative judgment. Although the post-Chernobyl patients are younger than the sporadic patients (although five out of 12 are older than 18), and often present lymph node metastases, the fact that the gene expression patterns are similar is a further argument suggesting that all these carcinomas represent the same disease.

Besides clinical similarities, some genetic differences have been reported between sporadic and chPTC. Whereas RET/PTC3 rearrangements dominate in post-Chernobyl carcinomas (Klugbauer *et al*, 1995; Nikiforov *et al*, 1997; Thomas *et al*, 1999; Santoro *et al*, 2000), both RAF mutations and RET/PTC1 or RET/PTC3 rearrangements are predominantly found in adult sporadic carcinomas. Recent findings revealed that BRAF mutations are usually rare in childhood thyroid carcinoma, both sporadic and radiation related, suggesting that thyroid carcinomas involving a BRAF mutation may have a longer latency period than those involving gene rearrangements (Kumagai *et al*, 2004; Lima *et al*, 2004). BRAF mutations and RET/PTC rearrangements are mutually exclusive in papillary carcinomas, both activating constitutively the RET/PTC–RAS–BRAF–MAPK pathway (Kimura *et al*, 2003; Soares *et al*, 2003). Of course, different levels of activity of this oncogenic pathway could lead to quantitative differences in growth rates and phenotypic expression. Our results suggest that genetic differences, RET/PTC-, BRAF-related, or others, between sporadic and chPTC are not mirrored by gene expression in the tumours. This supports the view that whatever the initial event, the phenotype of the tumour results from the transduction pathway activated, that is, in this case mainly the MAP kinase cascade, with different levels of activity (De Vita *et al*, 2000; Melillo *et al*, 2001). The cohort of post-Chernobyl patients, for which the timing of the cancer-causing event is precisely known, is a unique source of information for understanding the progression of naturally occurring cancers. Our clinical and therapeutical knowledge of sporadic papillary carcinomas can therefore be applied to the post-Chernobyl diseases and conversely. In particular, the measured delay between initial causing event and appearance of the disease in the post-Chernobyl patients provides a clue to estimate the timing of such events in sporadic carcinomas.

In conclusion, on the basis of their similar pathology, it is generally assumed that post-Chernobyl thyroid cancers are identical to sporadic papillary carcinomas. In this work, we investigated gene expression in both types of cancers by a variety of microarray methods and our analyses suggest that these gene expressions cannot be distinguished, although both tumours are clearly distinct from AAs. Post-Chernobyl, radiation-induced thyroid cancers and sporadic thyroid papillary carcinomas thus most likely represent the same disease. It does not exclude the possibility that further analysis will demonstrate that the post-Chernobyl carcinomas might belong to a more homogeneous restricted subset of carcinomas. This could even be expected owing to their similar induction mechanism, period of initiation and the similar age of patients of the investigated cohort.

## Figures and Tables

**Table 1:** Summary of the clinical parameters and gene alterations of the post-Chernobyl and sporadic papillary thyroid carcinomas studied

**Sample name**	**Age**	**Sex**	**Tumour size (cm)**	**Histological variant of PTC**	**Genotype**	**Regional LN metastasis**	**Distant metastasis**
*Post-Chernobyl*							
ch155	12	F	2.5	Solid	RET/PTC3	+	−
ch104	12	M	4.5	Papillary	RET/PTC3	+	−
ch163	11	M	4.5	Follicular	neg	+	−
ch12	14	F	2	Solid	RET/PTC3	+	+
ch70	18	F	5.5	Papillary	neg	+	+
ch109	13	F	7	Papillary	RET/PTC1	+	−
ch123	13	F	5	Follicular	neg	+	+
chs405	16	F	3	Papillary	RET/PTC	+	−
ch414	33	F	2.5	Follicular	neg	−	−
chs420	28	F	1.4	Follicular	neg	−	−
chs422	31	M	0.9	Follicular	BRAF^T1796A^	+ (few cells)	−
chs423	22	F	4.5	Papillary	neg	−	−
							
Sporadic:							
p1	51	M	2.5	Follicular	RET/PTC	+	−
p2	49	M	0.4	Papillary	ND	−	−
p3	59	M	6	Papillary	neg	+	−
p4	62	M	1	Papillary	BRAF^T1796A^	NA	NA
p5	45	F	3	Follicular	BRAF^T1796A^	−	−
p6	37	M	5	Papillary	ND	+	−
p11	37	F	1.5	Papillary	neg	+	−
p14	32	M	1.9	Follicular	RET/PTC	−	−

NA=not available; ND=not determined; neg=neither RET/PTC rearrangement nor BRAF mutation.

**Table 2:** Correlations between cDNA and Huang *et al* oligonucleotide microarray data (see text for details)

	**VIB sPTC**	**VIB chPTC**	**VIB AA**
*Correlations on all 6425 genes*			
Huang sPTC	0.49	0.64	0.03
			
*Correlations on the 50*% *most expressed genes*			
Huang sPTC	0.6	0.74	0.02
